# Crystal structure of 4-[(*E*)-(4-chloro­benzyl­idene)amino]-3-(2-methyl­benz­yl)-1*H*-1,2,4-triazole-5(4*H*)-thione

**DOI:** 10.1107/S1600536814018352

**Published:** 2014-08-16

**Authors:** Kamni, B. K. Sarojini, P. S. Manjula, B. Narayana, Sumati Anthal, Rajni Kant

**Affiliations:** a School of Physics, Shri Mata Vaishno Devi University, Katra 182 320, J&K, India; b Department of Chemistry, Mangalore University, Mangalagangotri 574 199, D.K., Mangalore, India; c Department of Chemistry, P A College of Engineering, Nadupadavu 574 153, D.K., Mangalore, India; d X-ray Crystallography Laboratory, Post-Graduate Department of Physics & Electronics, University of Jammu, Jammu Tawi 180 006, India

**Keywords:** crystal structure, Schiff base, 1,2,4-triazole, hydrogen bonding

## Abstract

In the title mol­ecule, C_17_H_15_ClN_4_S, the benzene rings form dihedral angles of 16.6 (1) and 77.2 (1)° with the triazole ring. The dihedral angle between the benzene rings is 86.6 (1)°. In the crystal, pairs of N—H⋯S hydrogen bonds form inversion dimers with graph-set notation *R*_2_^2^(8). Weak C—H⋯S hydrogen bonds link these dimers into layers parallel to (100). Weak intra­molecular C—H⋯S and C—H⋯N contacts are observed.

## Related literature   

For the chemistry of Schiff base compounds, see: Dubey & Vaid (1991 ▶); Yadav *et al.* (1994 ▶); Reddy & Lirgappa (1994 ▶); Wyrzykiewicz & Prukah (1998 ▶); Galic *et al.* (2001 ▶). For the biological activity of 1,2,4-triazole derivatives, see: Jones *et al.* (1965 ▶); Kane *et al.* (1988 ▶); Mullican *et al.* (1993 ▶); Cansiz *et al.* (2001 ▶). For the biological activity of sulfur- and nitro­gen-containing compounds, see: Malik *et al.* (2011 ▶); Wei & Bell (1982 ▶). For related structures, see: Ding *et al.* (2009 ▶); Vinduvahini *et al.* (2011 ▶); Almutairi *et al.* (2012 ▶); Sarojini *et al.* (2013 ▶, 2014*a* ▶,*b* ▶). For standard bond-lengths, see: Allen *et al.* (1987 ▶). For hydrogen-bond graph-set notation, see: Bernstein *et al.* (1995 ▶).
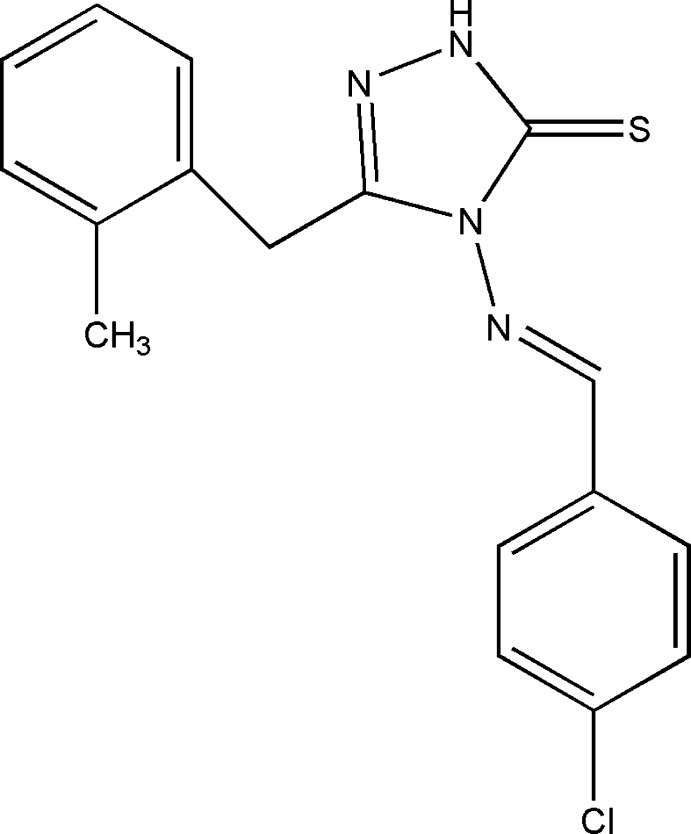


## Experimental   

### Crystal data  

C_17_H_15_ClN_4_S
*M* *_r_* = 342.84
Monoclinic, 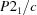

*a* = 13.4821 (9) Å
*b* = 6.8479 (4) Å
*c* = 18.4895 (12) Å
β = 100.443 (6)°
*V* = 1678.75 (18) Å^3^
*Z* = 4
Mo *K*α radiation
μ = 0.36 mm^−1^
*T* = 293 K
0.30 × 0.20 × 0.20 mm

### Data collection  

Oxford Diffraction Xcalibur, Sapphire3 diffractometer
Absorption correction: multi-scan (*CrysAlis PRO*; Oxford Diffraction, 2010 ▶) *T*_min_ = 0.858, *T*_max_ = 1.000
6947 measured reflections
3306 independent reflections
2252 reflections with *I* > 2σ(*I*)
*R*_int_ = 0.028

### Refinement  

*R*[*F* ^2^ > 2σ(*F* ^2^)] = 0.045
*wR*(*F* ^2^) = 0.115
*S* = 1.04
3306 reflections
213 parameters
H atoms treated by a mixture of independent and constrained refinement
Δρ_max_ = 0.20 e Å^−3^
Δρ_min_ = −0.24 e Å^−3^

### 

Data collection: *CrysAlis PRO* (Oxford Diffraction, 2010 ▶); cell refinement: *CrysAlis PRO*; data reduction: *CrysAlis PRO*; program(s) used to solve structure: *SHELXS97* (Sheldrick, 2008 ▶); program(s) used to refine structure: *SHELXL97* (Sheldrick, 2008 ▶); molecular graphics: *PLATON* (Spek, 2009 ▶) and *ORTEP-3 for Windows* (Farrugia, 2012 ▶); software used to prepare material for publication: *PLATON*.

## Supplementary Material

Crystal structure: contains datablock(s) I, New_Global_Publ_Block. DOI: 10.1107/S1600536814018352/lh5724sup1.cif

Structure factors: contains datablock(s) I. DOI: 10.1107/S1600536814018352/lh5724Isup2.hkl

Supporting information file. DOI: 10.1107/S1600536814018352/lh5724Isup3.cml

**Fig. 1.**
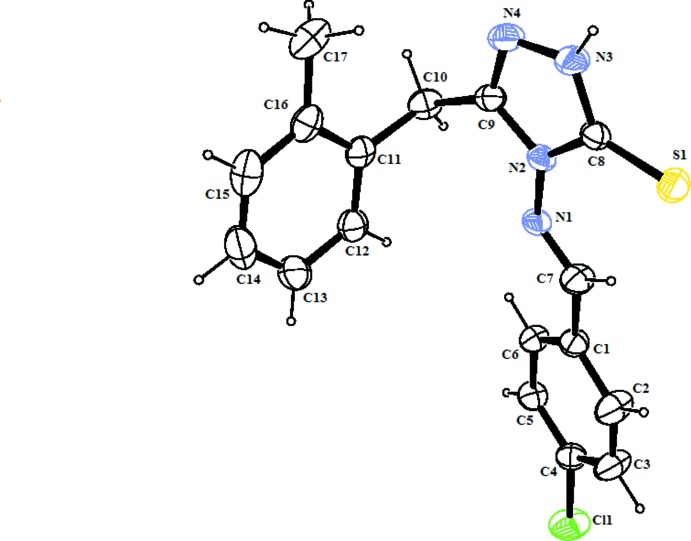
The molecular structure with ellipsoids drawn at the 40% probability level. H atoms are shown as small spheres of arbitrary radii.

. DOI: 10.1107/S1600536814018352/lh5724fig1.tif
The mol­ecular structure with ellipsoids drawn at the 40% probability level. H atoms are shown as small spheres of arbitrary radii.

**Fig. 2.**
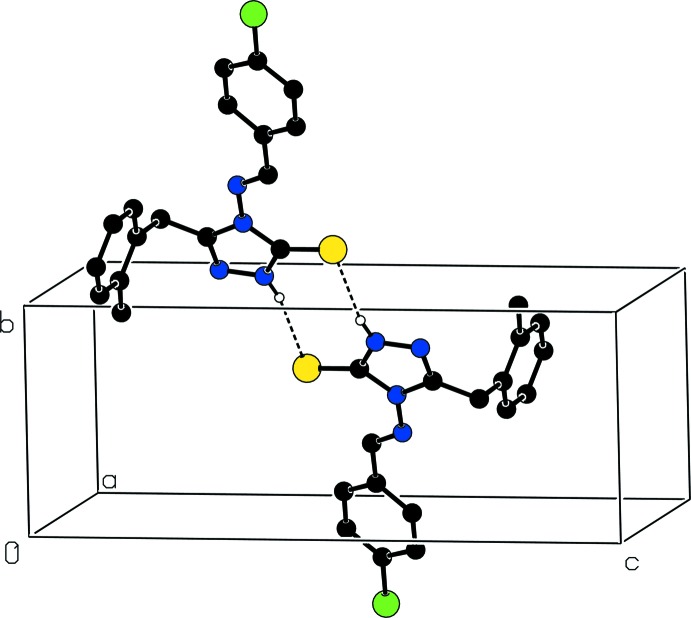
A pair molecules of the title compound. Dashed lines indicate N—H···S hydrogen bonds forming R22(8) graph set motifs linking the molecules into dimers.

**Fig. 3.**
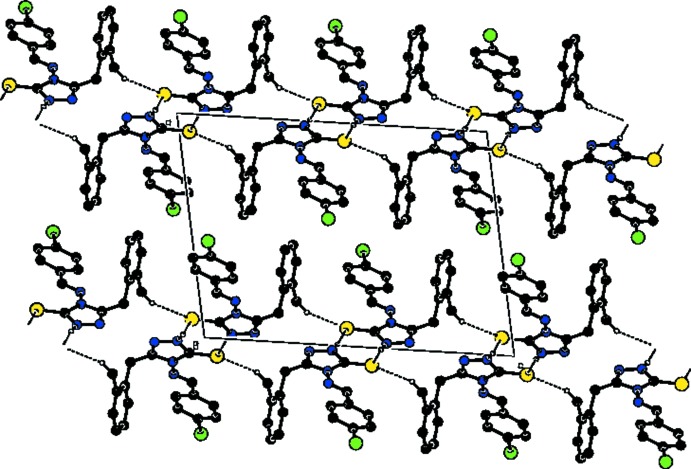
The packing arrangement of molecules viewed along the b axis. Hydrogen bonds are shown as dashed lines.

. DOI: 10.1107/S1600536814018352/lh5724fig2.tif
A pair mol­ecules of the title compound. Dashed lines indicate N—H⋯S hydrogen bonds forming 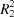
(8) graph set motifs linking the mol­ecules into dimers.

b . DOI: 10.1107/S1600536814018352/lh5724fig3.tif
The packing arrangement of mol­ecules viewed along the *b* axis. Hydrogen bonds are shown as dashed lines.

CCDC reference: 1018973

Additional supporting information:  crystallographic information; 3D view; checkCIF report

## Figures and Tables

**Table 1:** Hydrogen-bond geometry (Å, °)

*D*—H⋯*A*	*D*—H	H⋯*A*	*D*⋯*A*	*D*—H⋯*A*
N3—H3′⋯S1^i^	0.84 (3)	2.43 (3)	3.261 (2)	167 (2)
C17—H17*B*⋯S1^ii^	0.96	2.81	3.710 (3)	157
C17—H17*C*⋯N4	0.96	2.59	3.321 (4)	134
C7—H7⋯S1	0.93	2.45	3.203 (2)	138

Symmetry codes: (i) 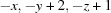; (ii) 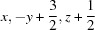.

## supplementary crystallographic information

### S1. Comment

The synthesis and structural investigation of Schiff base compounds have attracted much attention due to their interesting structures and potential applications. During the last few decades, there has been a considerable interest in the chemistry of Schiff base compounds (Dubey & Vaid 1991; Yadav *et al.*, 1994). The derivatives of 1,2,4-triazole are known to exhibit anti-inflammatory (Mullican *et al.*, 1993), antiviral (Jones *et al.*, 1965), antimicrobial (Cansiz *et al.*, 2001), and antidepressant activity (Kane *et al.*, 1988). Schiff bases, containing different donor atoms, also find use in analytical applications and metal coordination (Galic *et al.*, 2001; Wyrzykiewicz & Prukah, 1998; Reddy & Lirgappa, 1994). Since many compounds containing sulfur and nitrogen atoms are antihypertensive (Wei *et al.*, 1982) and fungicidal (Malik *et al.*, 2011), synthesis of the corresponding heterocyclic compounds could be of interest from the viewpoint of chemical reactivity and biological activity. The crystal structures of some of the related Schiff's bases *viz*: 3-Methyl-4-{(*E*)-[4-(methylsulfanyl)benzylidene]amino}-1*H*-1, 2,4-triazole-5(4*H*)-thione; 4-[(*E*)-(4-chloro&not;benzyl-idene)amino]-3-methyl-1H -1,2,4-triazole-5(4*H*)-thione; 4-[(*E*)-(4-Hy&not;droxy&not;benzyl&not;idene)amino] -3-methyl-1*H*-1,2,4-triazole-5(4*H*)-thione (Sarojini *et al.*, 2013, 2014*a*, *b*); 3-[2-(2,6-Dichloro-anilino)benzyl]-4- [(4-methoxybenzylidene)amino]-1*H*-1,2,4-triazole-5(4*H*) -thione (Vinduvahini *et al.*, 2011); 3-(Adamantan-1-yl)-1-[(4-ethylpiperazin-1-yl)methyl]-4- [(*E*)-(4-hydroxy-benzylidene)amino]-1*H*-1,2,4-triazole-5(4*H*) -thione (Almutairi *et al.*, 2012);(*E*)-4-[(4-Fluorobenzylidene)amino]-3-[1- (4-isobutylphenyl)ethyl]-1-(morpholinomethyl)-1*H*- 1,2,4-triazole-5(4*H*)-thione methanol hemisolvate, (*E*)-3-(2-ethoxyphenyl)-4-(2-fluorobenzylideneamino) -1*H*-1,2,4-triazole-5(4*H*)-thione (Ding *et al.*, 2009) have been reported. In the title compound (I) (Fig. 1), all bond lengths are within normal ranges (Allen *et al.*, 1987). The dihedral angles between the mean planes of the chloro-substituted benzene ring and methyl-substituted benzene ring is 86.6 (1)°. The triazole ring forms dihedral angles of 16.6 (1) and 77.2 (1)°, respectively, with the C1—C6 and C11—C16 rings. In the crystal, N—H···S hydrogen bonds and two intramolecular interactions of the type C—H···N and C—H···S are observed (Table 2). Centrosymmetric dimeric aggregates are formed by pairs of N3—H3'···S1 hydrogen bonds forming *R*_2_^2^(8) ring motifs (Bernstein *et al.*, 1995). Intermolecular C—H···S hydrogen bonds link the molecules into a two-dimensional network paralell to (100) (Fig. 3).

### S2. Experimental

4-Chlorobenzaldehyde (0.01 mol, 1.40 g) in ethanol (15 ml) was added to 4-amino-3-(2-methylbenzyl)-1*H*-1,2,4-triazole-5(4*H*)-thione (0.01 mol, 2.20 g) and heated to form a clear solution. To this few drops of conc. H_2_SO_4_ were added as a catalyst and refluxed for 36 h on water bath. The precipitate formed was filtered and recrystallized from methanol to get the title compound. Single crystals were obtained from methanol (mp. 443–445 K).

### S3. Refinement

H3' attached to N3 was located in a difference map and refined isotropically. The remaining H atoms were positioned geometrically and were treated as riding on their parent C atoms, with C—H distances of 0.93–0.97 A; and with *U*_iso_(H) = 1.2*U*_eq_(C), except for the methyl groups where *U*_iso_(H) = 1.5*U*_eq_(C).

### Crystal data

**Table:**

C_17_H_15_ClN_4_S	*F*(000) = 712
*M**_r_* = 342.84	*D*_x_ = 1.356 Mg m^−^^3^
Monoclinic, *P*2_1_/*c*	Mo *K*α radiation, λ = 0.71073 Å
Hall symbol: -P 2ybc	Cell parameters from 1855 reflections
*a* = 13.4821 (9) Å	θ = 3.6–26.5°
*b* = 6.8479 (4) Å	µ = 0.36 mm^−^^1^
*c* = 18.4895 (12) Å	*T* = 293 K
β = 100.443 (6)°	Block, white
*V* = 1678.75 (18) Å^3^	0.30 × 0.20 × 0.20 mm
*Z* = 4	

### Data collection

**Table:**

Oxford Diffraction Xcalibur, Sapphire3 diffractometer	3306 independent reflections
Radiation source: fine-focus sealed tube	2252 reflections with *I* > 2σ(*I*)
Graphite monochromator	*R*_int_ = 0.028
Detector resolution: 16.1049 pixels mm^-1^	θ_max_ = 26.0°, θ_min_ = 3.6°
ω scans	*h* = −16→12
Absorption correction: multi-scan (*CrysAlis PRO*; Oxford Diffraction, 2010)	*k* = −8→5
*T*_min_ = 0.858, *T*_max_ = 1.000	*l* = −22→18
6947 measured reflections	

### Refinement

**Table:**

Refinement on *F*^2^	Primary atom site location: structure-invariant direct methods
Least-squares matrix: full	Secondary atom site location: difference Fourier map
*R*[*F*^2^ > 2σ(*F*^2^)] = 0.045	Hydrogen site location: inferred from neighbouring sites
*wR*(*F*^2^) = 0.115	H atoms treated by a mixture of independent and constrained refinement
*S* = 1.04	*w* = 1/[σ^2^(*F*_o_^2^) + (0.0402*P*)^2^ + 0.2834*P*] where *P* = (*F*_o_^2^ + 2*F*_c_^2^)/3
3306 reflections	(Δ/σ)_max_ < 0.001
213 parameters	Δρ_max_ = 0.20 e Å^−^^3^
0 restraints	Δρ_min_ = −0.24 e Å^−^^3^

### Special details

**Table:**

Experimental. *CrysAlis PRO*, Oxford Diffraction Ltd., Version 1.171.34.40 (release 27–08-2010 CrysAlis171. NET) (compiled Aug 27 2010,11:50:40) Empirical absorption correction using spherical harmonics, implemented in SCALE3 ABSPACK scaling algorithm.
Geometry. All e.s.d.'s (except the e.s.d. in the dihedral angle between two l.s. planes) are estimated using the full covariance matrix. The cell e.s.d.'s are taken into account individually in the estimation of e.s.d.'s in distances, angles and torsion angles; correlations between e.s.d.'s in cell parameters are only used when they are defined by crystal symmetry. An approximate (isotropic) treatment of cell e.s.d.'s is used for estimating e.s.d.'s involving l.s. planes.
Refinement. Refinement of *F*^2^ against ALL reflections. The weighted *R*-factor *wR* and goodness of fit *S* are based on *F*^2^, conventional *R*-factors *R* are based on *F*, with *F* set to zero for negative *F*^2^. The threshold expression of *F*^2^ > σ(*F*^2^) is used only for calculating *R*-factors(gt) *etc*. and is not relevant to the choice of reflections for refinement. *R*-factors based on *F*^2^ are statistically about twice as large as those based on *F*, and *R*- factors based on ALL data will be even larger.

### Fractional atomic coordinates and isotropic or equivalent isotropic displacement parameters (Å^2^)

**Table:**

	*x*	*y*	*z*	*U*_iso_*/*U*_eq_	
S1	0.07980 (5)	0.72738 (9)	0.46649 (4)	0.0604 (2)	
Cl1	0.43301 (6)	−0.35687 (10)	0.54977 (5)	0.0791 (3)	
N2	0.11694 (13)	0.6086 (2)	0.61288 (11)	0.0416 (5)	
N1	0.17171 (13)	0.4355 (2)	0.61469 (11)	0.0444 (5)	
C6	0.27146 (17)	0.0685 (3)	0.61625 (13)	0.0454 (6)	
H6	0.2424	0.0961	0.6570	0.055*	
C9	0.09068 (17)	0.6762 (3)	0.67729 (13)	0.0450 (6)	
C1	0.25988 (17)	0.1987 (3)	0.55810 (13)	0.0468 (6)	
N4	0.03338 (15)	0.8286 (3)	0.66436 (12)	0.0576 (6)	
C7	0.20400 (19)	0.3820 (3)	0.55848 (15)	0.0550 (7)	
H7	0.1924	0.4596	0.5165	0.066*	
C4	0.36590 (18)	−0.1416 (3)	0.55312 (14)	0.0491 (6)	
C5	0.32542 (17)	−0.1010 (3)	0.61443 (14)	0.0474 (6)	
H5	0.3344	−0.1868	0.6541	0.057*	
N3	0.02267 (16)	0.8556 (3)	0.59013 (13)	0.0569 (6)	
C11	0.23908 (18)	0.6491 (3)	0.77823 (12)	0.0459 (6)	
C10	0.12992 (18)	0.5931 (3)	0.75064 (14)	0.0516 (6)	
H10A	0.1244	0.4520	0.7481	0.062*	
H10B	0.0890	0.6391	0.7853	0.062*	
C12	0.31449 (19)	0.5143 (4)	0.77442 (14)	0.0549 (7)	
H12	0.2972	0.3899	0.7562	0.066*	
C8	0.07340 (16)	0.7288 (3)	0.55585 (14)	0.0456 (6)	
C3	0.3549 (2)	−0.0174 (4)	0.49465 (15)	0.0663 (8)	
H3	0.3826	−0.0473	0.4534	0.080*	
C16	0.2652 (2)	0.8359 (4)	0.80624 (13)	0.0554 (7)	
C14	0.4396 (2)	0.7446 (5)	0.82462 (17)	0.0775 (9)	
H14	0.5069	0.7777	0.8407	0.093*	
C2	0.3021 (2)	0.1532 (4)	0.49789 (15)	0.0699 (8)	
H2	0.2948	0.2397	0.4585	0.084*	
C17	0.1871 (2)	0.9886 (4)	0.81350 (16)	0.0759 (9)	
H17A	0.2199	1.1030	0.8366	0.114*	
H17B	0.1412	0.9382	0.8430	0.114*	
H17C	0.1506	1.0221	0.7656	0.114*	
C15	0.3658 (3)	0.8780 (4)	0.82831 (16)	0.0738 (9)	
H15	0.3842	1.0020	0.8464	0.089*	
C13	0.4148 (2)	0.5614 (5)	0.79722 (15)	0.0720 (8)	
H13	0.4649	0.4701	0.7941	0.086*	
H3'	−0.0073 (19)	0.954 (4)	0.5690 (14)	0.064 (8)*	

### Atomic displacement parameters (Å^2^)

**Table:**

	*U*^11^	*U*^22^	*U*^33^	*U*^12^	*U*^13^	*U*^23^
S1	0.0735 (5)	0.0570 (4)	0.0470 (4)	0.0249 (3)	0.0007 (3)	−0.0027 (3)
Cl1	0.0901 (6)	0.0569 (4)	0.0945 (6)	0.0351 (4)	0.0281 (5)	0.0062 (4)
N2	0.0369 (10)	0.0382 (10)	0.0481 (12)	0.0069 (8)	0.0032 (9)	−0.0019 (9)
N1	0.0408 (11)	0.0388 (10)	0.0514 (13)	0.0099 (8)	0.0023 (10)	−0.0040 (9)
C6	0.0512 (14)	0.0440 (13)	0.0430 (15)	0.0035 (11)	0.0138 (12)	0.0008 (11)
C9	0.0403 (13)	0.0465 (13)	0.0502 (16)	0.0032 (10)	0.0135 (11)	−0.0024 (11)
C1	0.0506 (14)	0.0438 (13)	0.0451 (15)	0.0129 (10)	0.0062 (12)	0.0019 (11)
N4	0.0574 (13)	0.0605 (13)	0.0577 (15)	0.0195 (10)	0.0177 (11)	0.0014 (11)
C7	0.0651 (17)	0.0471 (14)	0.0531 (17)	0.0207 (12)	0.0120 (14)	0.0080 (12)
C4	0.0500 (14)	0.0422 (13)	0.0553 (16)	0.0108 (11)	0.0101 (12)	−0.0024 (12)
C5	0.0526 (15)	0.0406 (12)	0.0486 (15)	0.0067 (11)	0.0079 (12)	0.0093 (11)
N3	0.0616 (14)	0.0550 (13)	0.0536 (15)	0.0255 (11)	0.0095 (11)	0.0014 (11)
C11	0.0556 (15)	0.0528 (14)	0.0308 (13)	−0.0013 (11)	0.0119 (11)	0.0091 (11)
C10	0.0585 (16)	0.0488 (13)	0.0522 (16)	−0.0001 (12)	0.0223 (13)	0.0064 (12)
C12	0.0590 (17)	0.0611 (15)	0.0430 (16)	0.0058 (13)	0.0051 (13)	0.0030 (12)
C8	0.0397 (13)	0.0430 (12)	0.0517 (15)	0.0075 (10)	0.0021 (11)	−0.0046 (11)
C3	0.090 (2)	0.0651 (17)	0.0493 (17)	0.0296 (15)	0.0280 (15)	0.0052 (14)
C16	0.0769 (19)	0.0574 (15)	0.0338 (14)	−0.0077 (14)	0.0151 (13)	0.0048 (12)
C14	0.063 (2)	0.108 (3)	0.057 (2)	−0.0215 (19)	−0.0024 (16)	0.0029 (18)
C2	0.101 (2)	0.0657 (17)	0.0464 (17)	0.0376 (16)	0.0231 (16)	0.0186 (14)
C17	0.115 (3)	0.0588 (17)	0.059 (2)	0.0044 (16)	0.0285 (18)	−0.0072 (14)
C15	0.092 (2)	0.076 (2)	0.0499 (18)	−0.0228 (18)	0.0031 (17)	−0.0016 (15)
C13	0.0611 (19)	0.103 (2)	0.0496 (18)	0.0169 (17)	0.0025 (15)	0.0046 (16)

### Geometric parameters (Å, º)

**Table:**

S1—C8	1.670 (3)	C11—C12	1.384 (3)
Cl1—C4	1.736 (2)	C11—C16	1.401 (3)
N2—C8	1.382 (3)	C11—C10	1.517 (3)
N2—C9	1.382 (3)	C10—H10A	0.9700
N2—N1	1.394 (2)	C10—H10B	0.9700
N1—C7	1.252 (3)	C12—C13	1.380 (4)
C6—C5	1.374 (3)	C12—H12	0.9300
C6—C1	1.383 (3)	C3—C2	1.375 (3)
C6—H6	0.9300	C3—H3	0.9300
C9—N4	1.295 (3)	C16—C15	1.375 (4)
C9—C10	1.477 (3)	C16—C17	1.507 (4)
C1—C2	1.375 (3)	C14—C15	1.362 (4)
C1—C7	1.465 (3)	C14—C13	1.371 (4)
N4—N3	1.366 (3)	C14—H14	0.9300
C7—H7	0.9300	C2—H2	0.9300
C4—C3	1.363 (3)	C17—H17A	0.9600
C4—C5	1.373 (3)	C17—H17B	0.9600
C5—H5	0.9300	C17—H17C	0.9600
N3—C8	1.335 (3)	C15—H15	0.9300
N3—H3'	0.85 (2)	C13—H13	0.9300
			
C8—N2—C9	108.55 (18)	C11—C10—H10B	109.2
C8—N2—N1	132.44 (19)	H10A—C10—H10B	107.9
C9—N2—N1	118.78 (19)	C13—C12—C11	121.1 (3)
C7—N1—N2	119.4 (2)	C13—C12—H12	119.4
C5—C6—C1	120.7 (2)	C11—C12—H12	119.4
C5—C6—H6	119.6	N3—C8—N2	102.0 (2)
C1—C6—H6	119.6	N3—C8—S1	126.62 (18)
N4—C9—N2	110.4 (2)	N2—C8—S1	131.34 (17)
N4—C9—C10	125.6 (2)	C4—C3—C2	118.6 (2)
N2—C9—C10	123.8 (2)	C4—C3—H3	120.7
C2—C1—C6	118.6 (2)	C2—C3—H3	120.7
C2—C1—C7	119.1 (2)	C15—C16—C11	118.0 (3)
C6—C1—C7	122.3 (2)	C15—C16—C17	119.7 (3)
C9—N4—N3	104.2 (2)	C11—C16—C17	122.3 (3)
N1—C7—C1	120.7 (2)	C15—C14—C13	120.1 (3)
N1—C7—H7	119.6	C15—C14—H14	120.0
C1—C7—H7	119.6	C13—C14—H14	120.0
C3—C4—C5	121.8 (2)	C3—C2—C1	121.4 (2)
C3—C4—Cl1	119.0 (2)	C3—C2—H2	119.3
C5—C4—Cl1	119.28 (19)	C1—C2—H2	119.3
C4—C5—C6	118.9 (2)	C16—C17—H17A	109.5
C4—C5—H5	120.6	C16—C17—H17B	109.5
C6—C5—H5	120.6	H17A—C17—H17B	109.5
C8—N3—N4	114.8 (2)	C16—C17—H17C	109.5
C8—N3—H3'	123.0 (18)	H17A—C17—H17C	109.5
N4—N3—H3'	121.7 (18)	H17B—C17—H17C	109.5
C12—C11—C16	119.3 (2)	C14—C15—C16	122.4 (3)
C12—C11—C10	119.4 (2)	C14—C15—H15	118.8
C16—C11—C10	121.2 (2)	C16—C15—H15	118.8
C9—C10—C11	112.06 (19)	C14—C13—C12	119.1 (3)
C9—C10—H10A	109.2	C14—C13—H13	120.4
C11—C10—H10A	109.2	C12—C13—H13	120.4
C9—C10—H10B	109.2		
			
C8—N2—N1—C7	12.7 (3)	C10—C11—C12—C13	178.3 (2)
C9—N2—N1—C7	−173.4 (2)	N4—N3—C8—N2	2.0 (3)
C8—N2—C9—N4	0.5 (3)	N4—N3—C8—S1	−177.43 (18)
N1—N2—C9—N4	−174.75 (18)	C9—N2—C8—N3	−1.4 (2)
C8—N2—C9—C10	−175.1 (2)	N1—N2—C8—N3	172.9 (2)
N1—N2—C9—C10	9.7 (3)	C9—N2—C8—S1	177.92 (18)
C5—C6—C1—C2	1.1 (4)	N1—N2—C8—S1	−7.7 (4)
C5—C6—C1—C7	−179.2 (2)	C5—C4—C3—C2	0.3 (4)
N2—C9—N4—N3	0.7 (3)	Cl1—C4—C3—C2	−179.7 (2)
C10—C9—N4—N3	176.2 (2)	C12—C11—C16—C15	0.6 (3)
N2—N1—C7—C1	−178.63 (19)	C10—C11—C16—C15	−178.2 (2)
C2—C1—C7—N1	−174.1 (3)	C12—C11—C16—C17	−178.7 (2)
C6—C1—C7—N1	6.1 (4)	C10—C11—C16—C17	2.5 (3)
C3—C4—C5—C6	0.8 (4)	C4—C3—C2—C1	−0.7 (5)
Cl1—C4—C5—C6	−179.26 (18)	C6—C1—C2—C3	0.0 (4)
C1—C6—C5—C4	−1.5 (4)	C7—C1—C2—C3	−179.7 (3)
C9—N4—N3—C8	−1.7 (3)	C13—C14—C15—C16	0.9 (5)
N4—C9—C10—C11	−101.5 (3)	C11—C16—C15—C14	−0.8 (4)
N2—C9—C10—C11	73.5 (3)	C17—C16—C15—C14	178.5 (3)
C12—C11—C10—C9	−102.1 (2)	C15—C14—C13—C12	−0.7 (5)
C16—C11—C10—C9	76.6 (3)	C11—C12—C13—C14	0.5 (4)
C16—C11—C12—C13	−0.5 (4)		

### Hydrogen-bond geometry (Å, º)

**Table:**

*D*—H···*A*	*D*—H	H···*A*	*D*···*A*	*D*—H···*A*
N3—H3′···S1^i^	0.84 (3)	2.43 (3)	3.261 (2)	167 (2)
C17—H17*B*···S1^ii^	0.96	2.81	3.710 (3)	157
C17—H17*C*···N4	0.96	2.59	3.321 (4)	134
C7—H7···S1	0.93	2.45	3.203 (2)	138

Symmetry codes: (i) −*x*, −*y*+2, −*z*+1; (ii) *x*, −*y*+3/2, *z*+1/2.
