# Supplementary figures and images for: Crystal structure of 4-[(E)-(4-chloro­benzyl­idene)amino]-3-(2-methyl­benz­yl)-1H-1,2,4-triazole-5(4H)-thione
Source: Acta Crystallogr Sect E Struct Rep Online. 2014 Aug 16;70(Pt 9):o1015–6. doi: 10.1107/S1600536814018352 (PMC4186062; doi:10.1107/S1600536814018352)

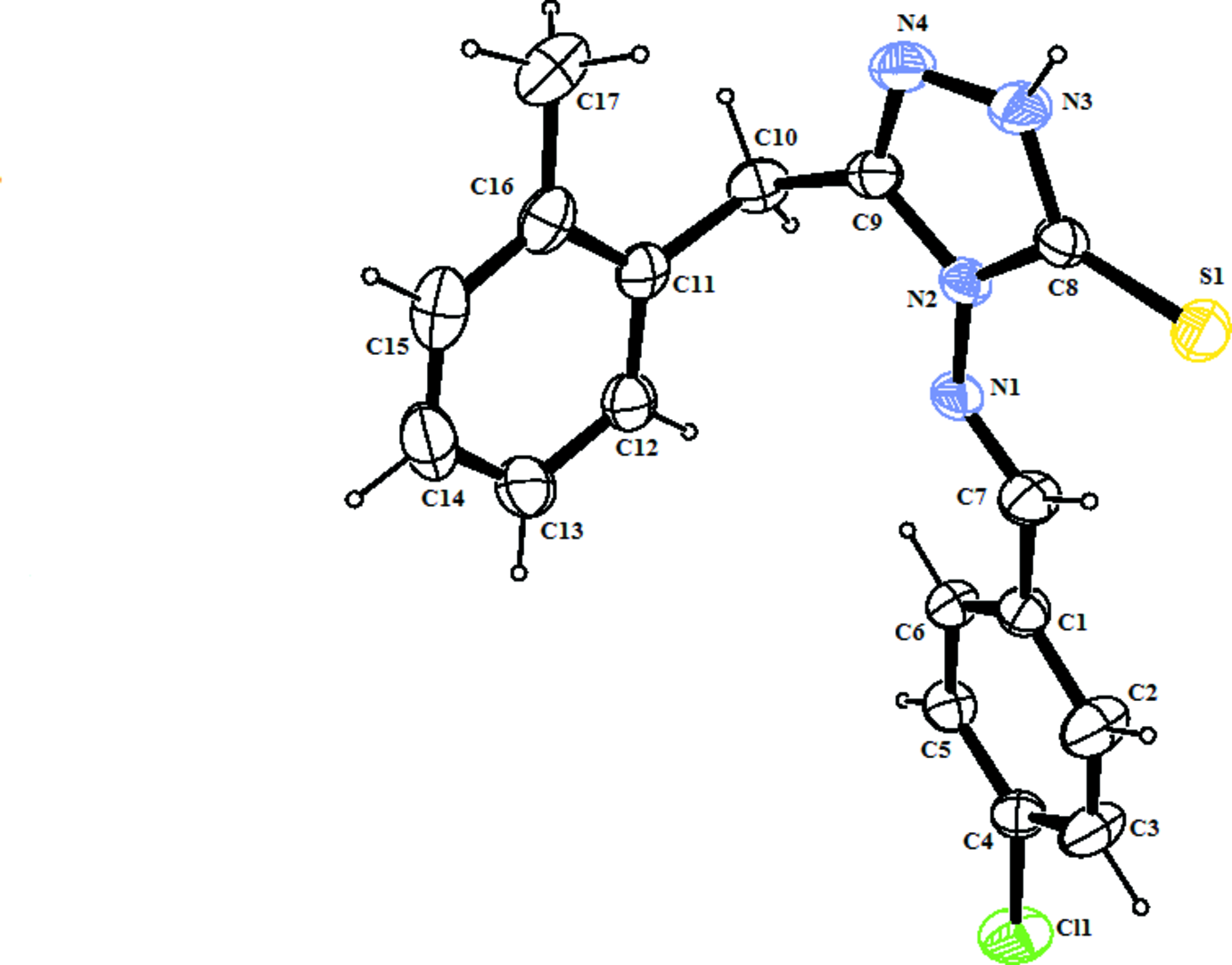

Supplement: Supplementary file 4 [file e-70-o1015-fig1.tif]

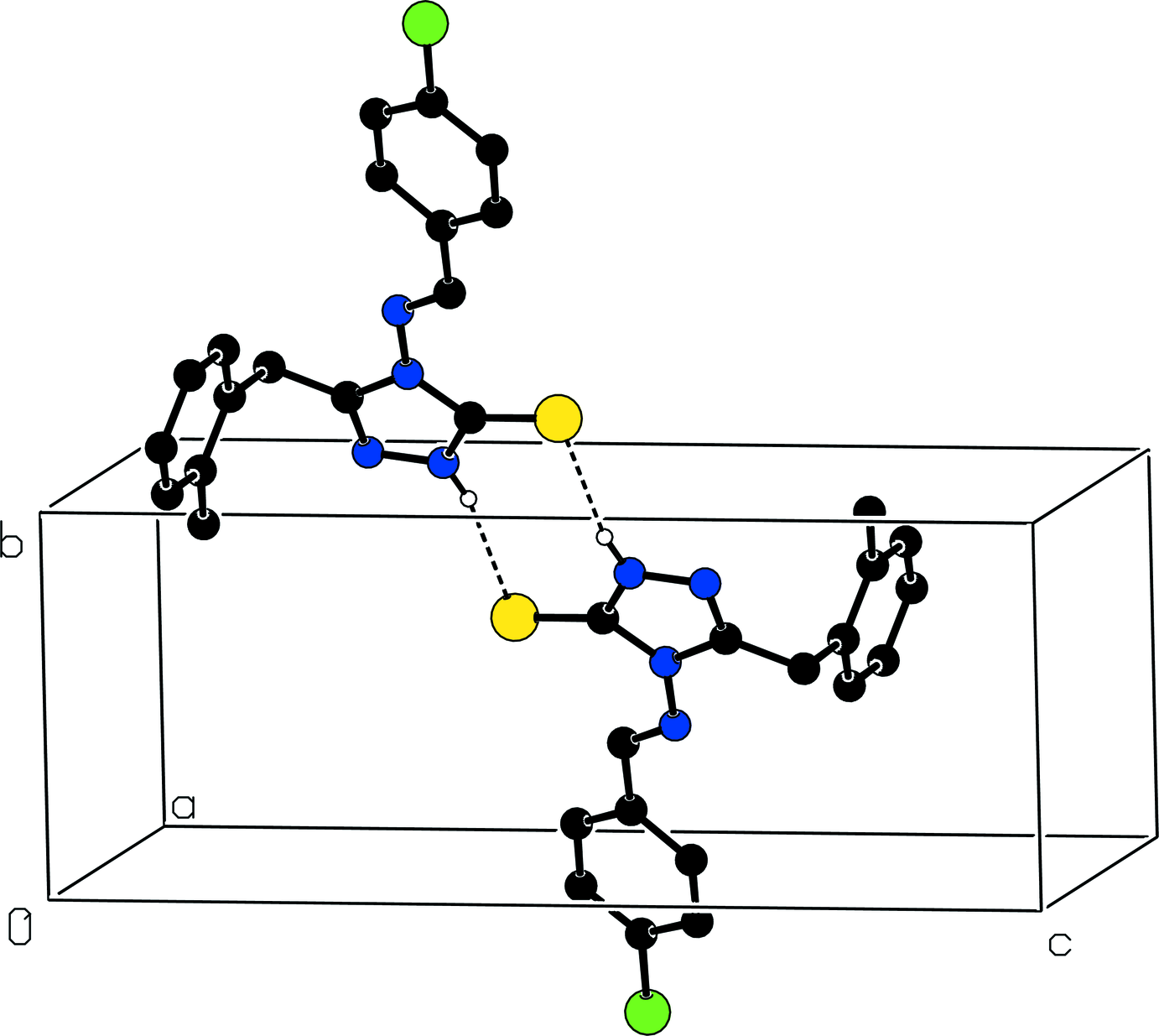

Supplement: Supplementary file 5 [file e-70-o1015-fig2.tif]

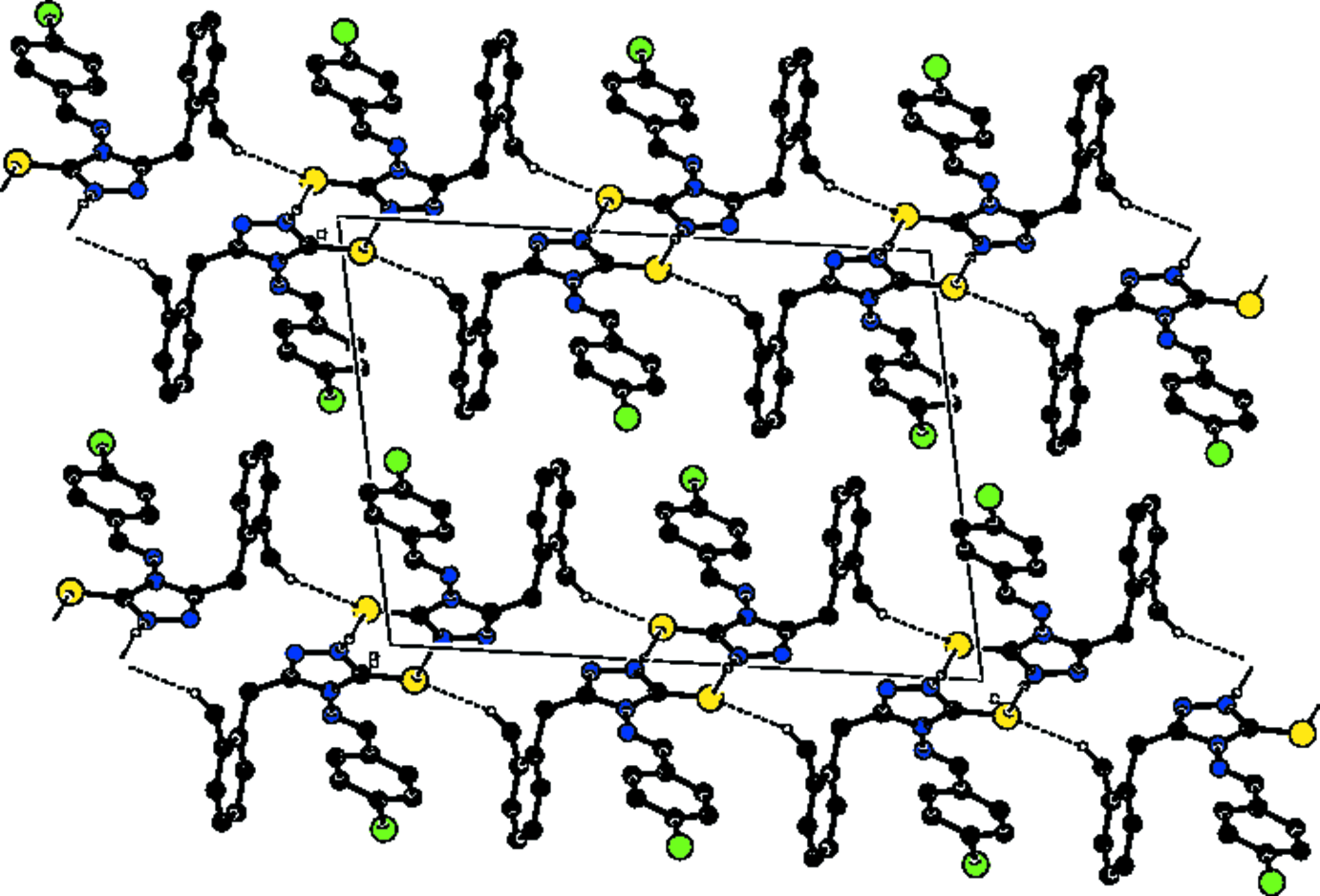

Supplement: Supplementary file 6 [file e-70-o1015-fig3.tif]
